# A Clustering-Based Machine Learning Approach for Mortality Prediction in Gastrointestinal Bleeding: Development and Validation

**DOI:** 10.1016/j.gastha.2026.100985

**Published:** 2026-04-24

**Authors:** Laith Alomari, Zaid Al-Fakhouri, Jaber Jaradat, Daniel Simadibrata, Ahmad Al-Riyalat, Justin Lam, Emmanuel Otabor, Yaman Jarrar, Abdallah Massad, Jana Alomari, Ala Abdel-Jalil, Ghideon Ezaz

**Affiliations:** 1 Department of Medicine, Jefferson Einstein Philadelphia Hospital, Philadelphia, Pennsylvania; 2 Department of Medicine, The MetroHealth System, Case Western Reserve University, Cleveland, Ohio; 3 Faculty of Medicine, Mu’tah University, Al-Karak, Jordan; 4 Department of Medicine, Lehigh Valley Health Network, Allentown, Pennsylvania; 5 Department of Medicine, University of Texas Medical Branch, Galveston, Texas; 6 Faculty of Medicine, Jordan University of Science and Technology, Irbid, Jordan; 7 Division of Gastroenterology and Hepatology, MetroHealth Medical Center, Case Western Reserve University, Cleveland, Ohio; 8 Division of Gastroenterology and Hepatology, Department of Medicine, Jefferson Einstein Philadelphia Hospital, Philadelphia, Pennsylvania

**Keywords:** Gastrointestinal Bleeding, Machine Learning, Mortality Prediction, Ensemble Model, Risk Stratification

## Abstract

**Background and Aims:**

Gastrointestinal bleeding (GIB) is a life-threatening emergency with considerable morbidity and mortality. Traditional risk scores like AIMS65 and Glasgow-Blatchford Score (GBS) are limited in capturing nonlinear clinical interactions. We developed and externally validated a machine learning model to predict 30-day mortality in GIB patients.

**Methods:**

We retrospectively analyzed 5453 emergency department patients with GIB from the Medical Information Mart for Intensive Care IV–Emergency Department database for model development, with external validation using 7166 patients from Jefferson Health. Sixteen clinical and laboratory variables were selected based on a literature review and clinical relevance. The development cohort was divided into training (80%) and internal validation (20%) sets. Survivors were partitioned into 24 clusters using K-means, with separate random forest models trained on each cluster, combined with all deceased cases. Performance was evaluated using the area under the receiver-operating characteristic curve, sensitivity, and specificity on the external validation set, then benchmarked against AIMS65 and the GBS.

**Results:**

The model achieved an area under the receiver-operating characteristic curve of 0.884 (95% confidence interval: 0.863–0.905) on internal validation and 0.882 (95% confidence interval: 0.863–0.900) on external validation, significantly outperforming AIMS65 (0.737) and GBS (0.768) (*P* < .001). At the optimal threshold, the model achieved 87.9% sensitivity and 74.3% specificity on the external validation cohort. At maximum sensitivity thresholds, the model maintained higher specificity (54.4%) than AIMS65 (29.7%) and GBS (17.0%) (*P* < .001). Clustering identified distinct phenotypes with mortality ranging from 0.6% to 15.3%. SHapley Additive exPlanations analysis identified age, albumin, hemodynamic parameters, and presenting hemoglobin and platelet count as key predictors.

**Conclusion:**

Our model provides superior risk stratification for 30-day mortality in GIB compared to conventional scores, with validated generalizability and potential for integration into electronic health record systems.

## Introduction

Gastrointestinal bleeding (GIB) is a common medical emergency, with an annual incidence of 378.4–397.5 per 100,000 population.^1^ Presentation includes hematemesis, melena, hematochezia, and signs of hemodynamic instability in severe cases. Prompt assessment of bleeding severity is crucial, as it directly influences management strategies and patient outcomes.^2^

Effective risk stratification is essential in guiding clinical decisions, including the need for inpatient vs outpatient care and the urgency of interventions. Traditional risk assessment tools, including the Glasgow-Blatchford Score (GBS) and AIMS65, are used to predict outcomes in GIB patients.^3^ However, these scores have limitations, including variable predictive accuracy and inability to account for the complex interplay of clinical variables in diverse patient populations. Advancements in machine learning (ML) offer opportunities to develop more sophisticated predictive models that analyze large datasets, identify intricate patterns, and enhance prognostic precision in GIB cases.^4^

Recent studies have demonstrated ML’s potential in predicting mortality among patients with GIB. A gradient-boosting model achieved an area under the receiver-operating characteristic curve (AUC) of 0.91, outperforming the GBS (AUC 0.88).^5^ Another electronic health record (EHR)-based ML model achieved an AUC of 0.92, surpassing existing clinical scores in identifying very-low-risk patients suitable for safe discharge.^6^

These findings underscore ML models’ potential to improve risk stratification in GIB by capturing complex, nonlinear relationships among clinical variables, facilitating more accurate and individualized risk assessments. By identifying high-risk patients requiring intensive interventions and low-risk patients suitable for outpatient management, ML models can optimize resource utilization and reduce unnecessary hospital admissions.

This study introduces and validates an ML algorithm that predicts mortality in patients presenting with GIB and compares its performance with established risk scores, such as AIMS65 and GBS, to assess its potential advantages in clinical application.

## Materials and Methods

### Data Source

This study utilized the Medical Information Mart for Intensive Care IV–Emergency Department (MIMIC-IV-ED) database,^7^ encompassing approximately 425,000 deidentified ED visits at Beth Israel Deaconess Medical Center between 2011 and 2019. It contains information regarding demographics, vital signs, laboratory measurements, medications, and diagnostic codes. MIMIC-IV-ED links to the MIMIC-IV critical care database^8^ through shared identifiers, enabling patient-level analyses across emergency and inpatient settings.

External validation was performed using data from Jefferson Health, a multicenter healthcare system in greater Philadelphia comprising multiple academic medical centers and community hospitals, with patients presenting to the ED with GIB symptoms between December 2005 and December 2024.

### Cohort and Variable Selection

Patients presenting to the ED with GIB were identified through screening of chief complaints using keyword extraction techniques with terms related to GIB (Supplementary Material). In total, 5453 patients were identified from MIMIC-IV, of whom 236 (4.32%) died within 30 days of presentation. The Jefferson Health cohort was identified using similar criteria. After applying the same exclusion criteria and handling missing data consistently with the development protocol, 7166 patients were included, with 338 deaths (4.72%) within 30 days.

Age, gender, initial vital signs (temperature, blood pressure, heart rate, respiratory rate, and oxygen saturation), and laboratory values, including complete blood count, basic metabolic panel, coagulation profiles, liver function tests, blood gas, and lactate levels, were extracted from both cohorts. All predictor variables were collected at the time of ED presentation: vital signs represent initial triage values, and laboratory values represent the first available results from tests ordered during the ED encounter. Pertinent comorbidities—myocardial infarction, congestive heart failure, liver disease, and malignancy—were identified using International Classification of Diseases (ICD)-9 and ICD-10 codes (Supplementary Table 1).

Variables with more than 50% missing values were excluded. For the remaining variables, overall missingness was low (<10% for most variables), and missing data were imputed using the mean value of each respective variable. The 4 categorical predictors (hematemesis, melena, liver disease, and malignancy) are binary indicators derived from keyword extraction or diagnostic codes with negligible missingness by design, and thus did not require imputation. The final model utilized 16 variables selected based on literature review, clinical plausibility, and established use in risk scores^4^^,^^6^^,^^9^, ^10^, ^11^, ^12^, ^13^, ^14^: age, heart rate, systolic blood pressure (SBP), hemoglobin, white blood cell count, platelet count, bicarbonate, blood urea nitrogen (BUN), creatinine, albumin, international normalized ratio (INR), lactate, presentation with hematemesis, presentation with melena, history of liver disease, and history of malignancy. The target outcome was 30-day mortality. Data extraction methods are detailed in the Supplementary Material.

### Data Partitioning

The MIMIC-IV cohort of 5453 patients (236 deceased, 5217 survivors) was split into training (80%) and internal validation (20%) sets via stratified sampling to preserve the mortality ratio, yielding 4362 training and 1091 internal validation patients. The training set was used for model development and hyperparameter tuning, while the internal validation set was reserved for threshold optimization and initial performance evaluation. The entire Jefferson Health cohort of 7166 patients (338 deceased, 6828 survivors) served as an independent external validation set, without retraining or recalibration.

### Outcome Definition

The primary outcome was all-cause mortality within 30 days of the index ED presentation. The 30-day period commenced at the time of ED triage. Death occurring within this window, regardless of patient disposition (inpatient, discharged, or transferred), was classified as a positive outcome. Mortality was ascertained from the date of death field in MIMIC-IV and from institutional medical records for the Jefferson Health cohort.

### Statistical Analysis

Categorical variables were compared between survivors and nonsurvivors using the chi-square test, with Fisher’s exact test applied when expected cell counts were fewer than 5. Continuous variables were compared using the independent-samples *t*-test or the Mann–Whitney U test as appropriate. For the ensemble model, 95% confidence intervals (CIs) were derived from 5-fold cross-validation, with metrics computed independently for each fold and 95% CIs calculated from the mean and standard error across folds using the t-distribution with 4 degrees of freedom. For AIMS65 and the GBS, 95% CIs on the external validation set were estimated via nonparametric bootstrap resampling (1000 iterations, percentile method). AUC comparisons between the ensemble model and each traditional score were performed using the DeLong test. Sensitivity and specificity comparisons between paired classifiers were assessed using McNemar’s test. Calibration of the ensemble model was evaluated using the Hosmer–Lemeshow goodness-of-fit test. *P* < .05 was considered statistically significant. All analyses were performed in Python 3.11 using scikit-learn, shap, and standard libraries. All scripts can be accessed at: https://github.com/laithomari/gi_bleeding_mortality_prediction for reproducibility.

### Model Development

#### Handling class imbalance

The disproportion between deceased and surviving patients, known as class imbalance, presents a significant challenge in predictive modeling. Traditional approaches to address this issue include oversampling—replicating minority-class instances or creating synthetic samples—and undersampling—removing a portion of the majority-class instances to achieve a more balanced dataset. While these mitigate bias, oversampling may introduce spurious patterns by generating data not originally present in the population, and undersampling risks discarding valuable majority-class information that could enhance model performance.^15^^,^^16^ To circumvent these limitations, we implemented a clustering-based approach that preserves the real-world distribution of surviving patients while systematically incorporating all minority-class data.

#### Clustering-based ensemble, hyperparameter tuning, and threshold selection

**Figure 1:**
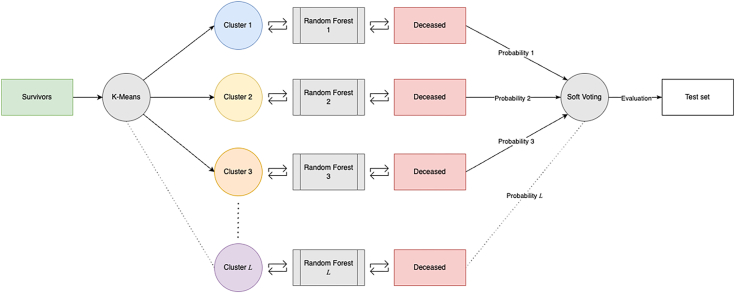
Schematic overview of the ensemble prediction methodology for mortality risk assessment in GI bleeding patients. The majority class (survivors) is partitioned into L (in our case, L = 24) clusters via K-means clustering, with each cluster subset combined with the minority class (deceased) to train and fine-tune individual random forest models. Probabilistic predictions from all L models are aggregated through soft voting, and the ensemble output is evaluated on the test set to generate final mortality risk predictions.

The majority-class (surviving) patients were partitioned into 24 clusters using K-means, based on the approximately 24:1 survivor-to-deceased ratio, ensuring nearly equal representation of both classes in each training subset. Each cluster was then combined with all minority-class (deceased) cases to form dedicated training subsets, allowing the classifier to learn localized decision boundaries for mortality within each region of the majority-class feature space. For each clustered subset, a random forest model was trained with its hyperparameters optimized using a 3-fold cross-validated randomized search to maximize the AUC. Hyperparameter search ranges and the final optimized hyperparameter values for each model are detailed in (Supplementary Table 2). The final prediction was then generated via *soft majority voting*, averaging predicted probabilities from all random forests to derive consensus mortality probability (Figure 1). This consensus probability was converted into a binary prediction at the threshold that maximized Youden’s J statistic (calculated as sensitivity + specificity − 1), determined using the internal validation set, and applied to the external validation cohort. This clustering-based ensemble approach addresses the challenges associated with traditional oversampling and undersampling techniques by maintaining the integrity of the original data distribution. Similar alternative data partitioning-based techniques have been proposed in the literature, demonstrating their effectiveness in handling class imbalance.^17^

### Model Evaluation

Performance was assessed using a 5-fold cross-validation on both internal and external validation cohorts. Key metrics included the AUC, sensitivity, specificity, positive predictive value (PPV), and negative predictive value (NPV). The clustering algorithm was also applied to the external validation cohort to analyze emergent clinical phenotypes and mortality patterns.

Two widely used GIB risk scores—AIMS65 and the GBS—were computed and evaluated on both cohorts. AIMS65 captures age, albumin, INR, SBP, and mental status, while GBS aggregates BUN, hemoglobin, blood pressure, and comorbid conditions. Binary thresholds (score ≥2 for AIMS65, score ≥6 for GBS) categorized high-risk patients, with predictions compared directly to the clustering-based ensemble.

### Model Interpretability

Global interpretability was pursued using SHapley Additive exPlanations (SHAP), a game-theoretic framework widely employed to decompose model predictions into feature-level contributions.^18^ SHAP values calculated across all test samples generated a global summary plot illustrating relative predictor importance in determining mortality risk, providing insight into the model’s decision-making process without relying on instance-level analyses. SHAP values have been widely adopted in medical ML models to interpret complex predictions.^19^, ^20^, ^21^, ^22^, ^23^

### Ethical Considerations

The MIMIC-IV and MIMIC-IV-ED databases comprise deidentified patient information, ensuring Health Insurance Portability and Accountability Act compliance. The Jefferson Health external validation data were obtained in a deidentified fashion under institutional data use agreements. Analyses with these datasets are classified as not involving human subjects research, obviating institutional review board approval. Dataset use aligns with the Declaration of Helsinki principles, as data are anonymized to protect patient confidentiality while facilitating medical research.

## Results

### Baseline Characteristics

Nonsurvivors were significantly older (69.4 vs 60.9 years, *P* < .001) with greater hemodynamic instability (elevated heart rate, lower blood pressure), more severe anemia, thrombocytopenia, and coagulopathy (Table 1). Laboratory findings revealed elevated BUN, lactate, and white blood cell counts, alongside lower albumin and bicarbonate levels in nonsurvivors (all *P* < .001). Hematemesis was more common in nonsurvivors (*P* < .001), while melena showed no difference. Nonsurvivors had a higher prevalence of liver disease, malignancy, and cardiac comorbidities (*P* < .001).

**Table 1:** Baseline Characteristics and Clinical Variables of Patients Grouped by 30-Day Mortality Status in the Development Cohort

Variable	Survivors; mean ± 95% CI or count (%)	Deceased; mean ± 95% CI or count (%)	*P* value
Demographics			
Age	61.87 ± (61.41–62.34)	69.35 ± (67.38–71.31)	<.0001
Gender (male)	2795 (53.57%)	139 (58.90%)	.1241
Vital signs (first recorded set)			
Temperature	97.95 ± (97.88–98.02)	95.55 ± (93.88–97.21)	.0232
Heart rate	84.78 ± (84.28–85.29)	92.70 ± (89.93–95.46)	<.0001
Respiratory rate	17.60 ± (17.53–17.66)	18.87 ± (18.39–19.35)	<.0001
Oxygen saturation	98.19 ± (98.12–98.27)	97.74 ± (97.37–98.10)	.0511
Systolic blood pressure	128.62 ± (128.00–129.25)	116.70 ± (113.57–119.82)	<.0001
Diastolic blood pressure	71.53 ± (71.00–72.07)	66.29 ± (64.01–68.58)	<.0001
Laboratory variables (first recorded)			
Hemoglobin	10.65 ± (10.58–10.72)	9.02 ± (8.70–9.33)	<.0001
Hematocrit	32.72 ± (32.51–32.92)	28.14 ± (27.23–29.06)	<.0001
WBC	9.06 ± (8.92–9.20)	12.55 ± (11.33–13.77)	<.0001
Platelets	227.98 ± (224.94–231.02)	203.98 ± (186.68–221.29)	<.0001
Sodium	138.59 ± (138.48–138.70)	137.16 ± (136.38–137.94)	<.0001
Potassium	4.36 ± (4.34–4.38)	4.66 ± (4.52–4.81)	<.0001
Chloride	102.17 ± (102.03–102.31)	100.20 ± (99.23–101.16)	<.0001
Bicarbonate	23.91 ± (23.81–24.01)	20.96 ± (20.19–21.73)	<.0001
BUN	25.75 ± (25.21–26.30)	40.81 ± (36.78–44.83)	<.0001
Creatinine	1.31 ± (1.27–1.35)	1.83 ± (1.60–2.05)	<.0001
Glucose	130.16 ± (128.42–131.90)	146.78 ± (133.03–160.54)	.0059
Anion gap	15.33 ± (15.22–15.43)	18.74 ± (17.79–19.70)	<.0001
Albumin	3.68 ± (3.65–3.70)	2.88 ± (2.77–2.98)	<.0001
PTT	31.98 ± (31.70–32.25)	38.96 ± (35.89–42.04)	<.0001
PT	16.11 ± (15.77–16.46)	20.82 ± (18.38–23.27)	<.0001
INR	1.48 ± (1.45–1.51)	1.86 ± (1.66–2.06)	<.0001
Fibrinogen	257.65 ± (239.75–275.54)	221.36 ± (187.92–254.80)	.0125
D-dimer	Insufficient data	Insufficient data	Insufficient data
Lactate	2.09 ± (2.04–2.15)	4.32 ± (3.69–4.94)	<.0001
pH	7.37 ± (7.36–7.37)	7.29 ± (7.26–7.32)	.0004
pCO2	40.64 ± (39.83–41.46)	42.50 ± (39.38–45.62)	.9638
CRP	34.87 ± (28.75–40.99)	83.72 ± (39.28–128.17)	.0088
ALT	35.54 ± (31.53–39.55)	67.15 ± (44.75–89.56)	<.0001
AST	60.03 ± (42.29–77.77)	177.08 ± (90.89–263.28)	<.0001
ALP	104.94 ± (101.87–108.02)	184.41 ± (142.82–226.00)	<.0001
Amylase	108.12 ± (49.30–166.95)	125.50 ± (−44.34 to 295.34)	.4911
Bilirubin, total	1.03 ± (0.96–1.10)	3.59 ± (2.49–4.70)	<.0001
Bilirubin, indirect	1.80 ± (1.55–2.06)	2.17 ± (1.34–3.01)	.732
Bilirubin, direct	1.99 ± (1.47–2.52)	6.72 ± (2.72–10.72)	.0001
Presenting symptoms			
Altered mental status	282 (5.41%)	13 (5.51%)	1
Hematemesis	957 (18.34%)	75 (31.78%)	<.0001
Melena	1525 (29.23%)	56 (23.73%)	.0803
Comorbidities			
Malignancy	556 (10.66%)	98 (41.53%)	<.0001
Metastasis	189 (3.62%)	58 (24.58%)	<.0001
Liver disease	871 (16.70%)	77 (32.63%)	<.0001
Acute MI	485 (9.30%)	41 (17.37%)	<.0001
Congestive heart failure	908 (17.40%)	69 (29.24%)	<.0001

Continuous variables are reported as mean ± 95% CI and compared using the independent-samples *t*-test or Mann–Whitney *U* test. Categorical variables are reported as count (%) and compared using the chi-square or Fisher’s exact test. Bold indicates inclusion in the final model.

ALP, alkaline phosphatase; ALT, alanine aminotransferase; AST, aspartate aminotransferase; BUN, blood urea nitrogen; CRP, C-reactive protein; MI, myocardial infarction; PT, prothrombin time; PTT, partial thromboplastin time.

### Cluster Description

**Figure 2:**
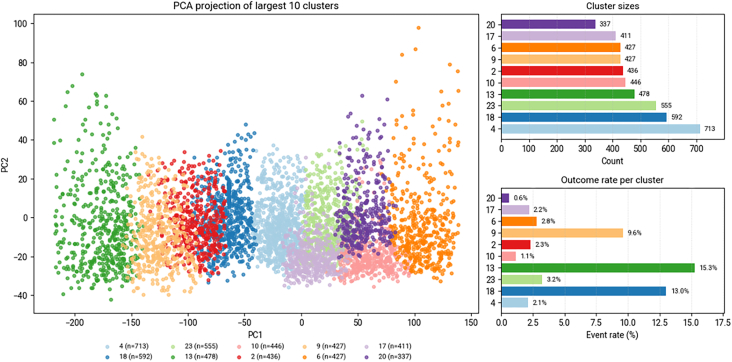
Principal component analysis (PCA) visualization and mortality distribution of patient clusters in the external validation cohort. The left panel shows PCA projection of the 10 largest clusters, with each cluster represented by a distinct color and demonstrating clear separation in the feature space. Right panels display cluster sizes (top) and corresponding 30-day mortality rates (bottom) for each cluster. Numbers in parentheses indicate cluster ID and sample size.

Clustering identified distinct phenotypes with mortality ranging from 0.60% to 15.30% (Figure 2). The highest-risk cluster (cluster 13, mortality 15.30%) comprised patients with advanced liver disease (31.20% prevalence), severe thrombocytopenia (platelet count 42 × 10^9^/L), coagulopathy (INR 1.79), marked hypoalbuminemia (2.81 g/dL), and frequent hematemesis (43.10%), suggesting possible variceal bleeding. On the other end, cluster 10 (mortality 1.10%) represented young patients (mean age 39 years) with preserved hemoglobin (12.7 g/dL) and minimal comorbidities. Clusters with severe renal dysfunction (clusters 0 and 22) demonstrated elevated creatinine (>4.9 mg/dL) and BUN (>94 mg/dL) with intermediate mortality rates (7.70%–7.80%). This phenotypic heterogeneity enabled cluster-specific mortality prediction, capturing complex interactions missed by uniform risk assessment (Supplementary Table 3).

### Model Performance

The ensemble model achieved an AUC of 0.882 (95% CI: 0.863–0.899) on external validation, closely matching internal validation (0.884, 95% CI: 0.863–0.905). At the optimal threshold of 0.852, the model demonstrated a sensitivity of 87.92%, a specificity of 74.27%, and an NPV of 99.20%, confirming its utility for identifying low-risk patients suitable for outpatient management. The Hosmer–Lemeshow goodness-of-fit test yielded a statistically significant result (χ^2^ = 36,557.5, df = 8, *P* < .001); however, this test is known to be overly sensitive in large cohorts (n = 7166), where even trivially small deviations from perfect calibration reach statistical significance.

In comparison, AIMS65 (threshold ≥2) achieved an AUC of 0.737 with a sensitivity of 71.89% and a specificity of 65.98%. The GBS (threshold ≥6) demonstrated an AUC of 0.768 with higher sensitivity (94.38%) but substantially lower specificity (39.87%).

**Figure 3:**
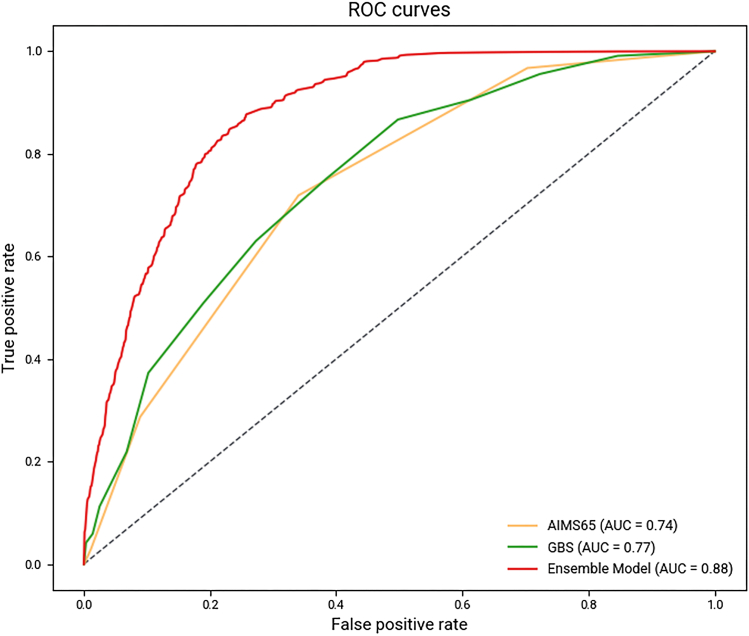
Receiver-operating characteristic (ROC) curves comparing the ensemble model, AIMS65, and Glasgow-Blatchford Score (GBS). The ensemble model demonstrated superior discrimination with an AUC of 0.88, compared to 0.74 for AIMS65 and 0.77 for GBS. The dashed diagonal line represents a random classifier.

The ensemble model significantly outperformed both traditional scores (*P* < .001 for both, Figure 3). The improved specificity vs GBS prevented 2409 false positives among 6828 survivors, substantially reducing unnecessary interventions. The ensemble's PPV (14.43%) exceeded both AIMS65 (9.47%) and GBS (7.21%) despite a low overall event rate (Table 2).

**Table 2:** Comparison of Performance Metrics Between the Ensemble Model (Threshold ≥0.852), AIMS65 (Threshold ≥2), and GBS (Threshold ≥6) on the External Validation Cohort

Metric	Ensemble model (mean ± 95% CI)	AIMS65 (mean ± 95% CI)	GBS (mean ± 95% CI)
AUC	0.8815 (0.8631–0.8999)	0.7368 (0.7159–0.7616)	0.7679 (0.7468–0.7908)
Sensitivity (recall)	0.8792 (0.8327–0.9256)	0.7189 (0.6739–0.7681)	0.9438 (0.9184–0.9680)
Specificity	0.7427 (0.7386–0.7468)	0.6598 (0.6489–0.6716)	0.3987 (0.3869–0.4102)
PPV (precision)	0.1443 (0.1184–0.1702)	0.0947 (0.0842–0.1065)	0.0721 (0.0644–0.0797)
NPV	0.9920 (0.9882–0.9958)	0.9793 (0.9752–0.9836)	0.9931 (0.9898–0.9960)

Metrics are reported as mean (95% CI). AUC comparisons used the DeLong test (both *P* < .001). Sensitivity and specificity comparisons used McNemar’s test: ensemble vs AIMS65 sensitivity *P* < .001, specificity *P* < .001; ensemble vs GBS sensitivity *P* = .689, specificity *P* < .001. Hosmer–Lemeshow calibration: χ^2^ = 36,557.5, df = 8, *P* < .001.

### Model Interpretability

**Figure 4:**
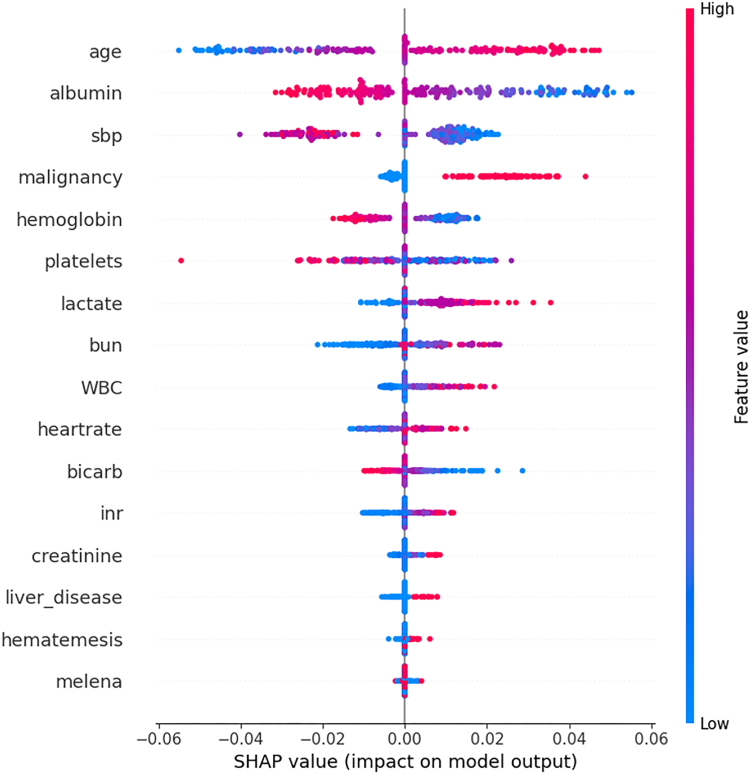
SHAP summary plot showing the impact of individual features on the predictions of the ensemble model. Features are ranked by their average magnitude of SHAP values. Each dot represents a patient, colored by the feature value (blue: low, pink: high).bicarb, serum bicarbonate.

SHAP analysis identified age and albumin as the most influential mortality predictors (Figure 4), followed by SBP, malignancy, hemoglobin, and platelet count. Additional important features included lactate (tissue hypoperfusion), BUN (renal dysfunction and upper GIB), and WBC (systemic inflammation). Liver disease and hematemesis showed significant impact within specific clusters, reflecting the model's capture of phenotype-specific risk patterns rather than uniform weights across all patients.

### Comparative Performance at Maximum Sensitivity Thresholds

To prioritize detection of at-risk patients, thresholds were selected to achieve near-maximal sensitivity. AIMS65 (threshold ≥1) achieved 96.75% sensitivity, GBS (threshold ≥2) achieved 99.41% sensitivity, and the ML model (threshold 0.845) achieved 98.34% sensitivity—beyond which further threshold reductions yielded negligible gains (<0.5%) with significant specificity loss.

At these thresholds, the ML model demonstrated a specificity of 54.4% (95% CI: 53.6–55.1), significantly outperforming AIMS65 (29.70%) and GBS (16.90%) (*P* < .001 for both), translating to 252 and 380 fewer false alarms per 1000 patients, respectively. While all tools exhibited strong NPV (>99%), the model improved PPV by approximately 50%–70% over traditional scores, positioning it as a pragmatic tool for balancing safety and efficiency in high-volume emergency settings (Table 3).

**Table 3:** Comparative Performance of the Machine Learning Model, AIMS65, and GBS at Maximum Sensitivity Thresholds on the External Validation Cohort

Metric	Ensemble model	AIMS65 (at ≥1 threshold)	GBS (at ≥2 threshold)
Sensitivity	0.9834 (0.9692–0.9977)	0.9675 (0.9461–0.9854)	0.9941 (0.9847–1.0000)
Specificity	0.5437 (0.5362–0.5511)	0.2967 (0.2868–0.3077)	0.1699 (0.1606–0.1789)
Positive predictive value (PPV)	0.0963 (0.0788–0.1137)	0.0638 (0.0572–0.0706)	0.0560 (0.0502–0.0619)
Negative predictive value (NPV)	0.9984 (0.9970–0.9998)	0.9946 (0.9910–0.9975)	0.9983 (0.9956–1.0000)
False positives (FP)	3116	4802	5668
False negatives (FN)	6	11	2

Metrics are reported as mean (95% CI). Specificity comparisons used McNemar’s test: ensemble vs AIMS65 *P* < .001; ensemble vs GBS *P* < .001. Sensitivity differences were not statistically significant at these near-maximal thresholds (ensemble vs AIMS65 *P* = .267; ensemble vs GBS *P* = .289).

## Discussion

This study presents a novel clustering-based ensemble ML model for predicting 30-day mortality in patients presenting with GIB, validated on an independent external cohort. The model achieved an AUC of 0.88 in external validation, outperforming AIMS65 (AUC 0.74) and GBS (AUC 0.77). By leveraging various clinical variables and a clustering approach that identifies distinct patient phenotypes, the model achieves more refined and individualized risk stratification compared to traditional linear scoring systems.

Traditional risk stratification tools rely on linear combinations of predefined variables, inadequately capturing complex, nonlinear interactions inherent to GIB outcomes. ML models excel at identifying intricate patterns and dynamic relationships among variables, such as synergistic effects between hemodynamic instability and coagulopathy or threshold-dependent interactions between age and comorbidities. For instance, while elevated BUN is linearly weighted in traditional scores, ML algorithms can discern how its prognostic significance varies nonlinearly with hemoglobin levels or platelet counts. This aligns with prior studies demonstrating ML's superiority in modeling heterogeneous clinical data, where outcomes are influenced by multifactorial, interdependent processes.^6^^,^^9^^,^^10^

Our model's performance (AUC 0.88) aligns closely with Shung et al’s ML model (AUC 0.92) while addressing critical limitations.^6^ First, their model incorporated 106 variables, including parameters lacking clear prognostic relevance to GIB, such as vitreous body disorders or vitamin B1 supplementation—factors unlikely to inform acute management decisions.^24^ Our 16-variable framework focuses on parameters routinely assessed during initial ED evaluations (eg, hemoglobin, vital signs, and coagulation studies), ensuring seamless clinical workflow integration. Second, their composite outcome included red blood cell transfusion—a protocolized decision based on hemoglobin thresholds (≤7 g/dL).^25^^,^^26^ This introduces confounding, as transfusion eligibility is directly linked to an input variable, artificially inflating associations between predictors and outcomes. By focusing solely on 30-day mortality, our model avoids combining protocol-driven interventions with intrinsic patient risk, enhancing prognostic validity. Finally, at near-maximal sensitivity thresholds (98.30%), our model achieved superior specificity (54.40%) using fewer variables, reducing over-triage and unnecessary admissions—crucial for cost-effective care in high-volume EDs.

A key feature of our study is the clustering-based ensemble framework, which addresses class imbalance while preserving real-world data integrity. Traditional approaches like oversampling or undersampling risk introducing synthetic artifacts or discarding informative majority-class samples. By partitioning the majority class (survivors) into 24 clusters via K-means—a number proportional to the observed class imbalance ratio—the clustering algorithm generates distinct patient groups with shared characteristics. External validation demonstrated effectiveness, identifying clusters with mortality rates ranging from 0.60% to 15.30%. Each cluster was paired with all minority-class (deceased) cases to train specialized random forest models, enabling individualized focus on mortality predictors within specific phenotypic subgroups. During ensemble aggregation, these models collectively captured multidimensional risk patterns, such as how age modulates the prognostic significance of coagulopathy or how malignancy exacerbates hemodynamic instability. This approach mitigates bias and leverages heterogeneity to enhance predictive granularity—a strategy not previously employed in GIB mortality models. Furthermore, by open-sourcing our framework, we enable dynamic adaptation to institutional needs: the number of clusters can be recalibrated to match local outcome distributions, and thresholds can be tailored to align with clinical priorities, ensuring generalizability and context-specific optimization.

Interpretability is critical for fostering clinician trust and ensuring actionable insights.^27^ SHAP analysis identified age, albumin, SBP, malignancy, hemoglobin, platelet count, lactate, BUN, and WBC as the most influential mortality predictors. Advanced age and malignancy align with their significance in the Rockall score.^28^ Low SBP reflects hemodynamic compromise central to AIMS65.^9^ Elevated lactate reflects tissue hypoperfusion and shock. Thrombocytopenia and hypoalbuminemia reflect coagulopathy, nutritional deficits, and advanced liver disease. BUN and hemoglobin are cornerstones of GBS. Hematemesis, while lower in overall importance, showed substantial impact within specific clusters, reaffirming its link to upper GIB severity.^29^, ^30^, ^31^ Elevated WBC underscores systemic inflammation’s prognostic relevance in GIB.^12^^,^^13^^,^^32^ All top predictors have been validated in prior studies or integrated into conventional risk scores, ensuring clinical plausibility while advancing predictive precision.

The model's simple architecture positions it for seamless EHR integration. Variables like hemoglobin, BUN, creatinine, and bicarbonate are embedded in standardized ED order sets, enabling automated data extraction without additional clinician input. This contrasts with complex ML models that depend on delayed measurements, hindering real-world adoption.^33^ By leveraging real-time EHR data, the model could generate automated risk alerts during triage, flagging high-risk patients for early care escalation while reassuring clinicians about the safety of outpatient management for low-risk cases.

The model's adaptability further enhances its translational potential. Institutions can recalibrate cluster numbers or decision thresholds to align with local demographics and resources. Centers serving older populations might prioritize sensitivity to avoid missed high-risk cases, while high-volume EDs could optimize specificity to reduce over-triage. This mirrors successful adaptive ML implementations in sepsis and cardiovascular risk prediction, where dynamic thresholds improved protocol adherence and resource allocation.^34^ These features position the model as a pragmatic, scalable solution for improving GIB outcomes while mitigating operational inefficiencies.

Nevertheless, several limitations merit consideration. First, reliance on static variables overlooks dynamic clinical trajectories, such as responses to resuscitation or evolving laboratory trends. Incorporating time-series data (eg, repeated vital signs, lactate clearance) could enhance accuracy, as demonstrated in critical care studies.^35^ Second, the relatively low mortality rate (4.70%) and small number of events per cluster may limit discernment of risk patterns in rare subgroups or patients with uncommon comorbidities. Larger prospective studies are needed to refine predictions in these populations. Additionally, ethical deployment requires rigorous algorithmic bias evaluation. Unmeasured confounders, such as socioeconomic status or healthcare access, may disproportionately influence outcomes in marginalized groups.^36^ Future work should integrate fairness-aware algorithms and validate performance across demographic subgroups. Furthermore, our exclusion of variables with greater than 50% missingness, while necessary to ensure data quality, may have removed predictors with potential prognostic value and represents a potential source of bias. Importantly, the dataset did not include key demographic variables such as race and ethnicity, which limits our ability to fully assess the model’s external validity and generalizability across diverse patient populations; this represents a major limitation that should be addressed in future validation studies.

Building on these findings, several avenues warrant exploration. First, real-time EHR integration could be tested in trials assessing impact on outcomes like time-to-intervention or mortality, comparing EDs using the model against those using standard risk scores. Second, incorporating dynamic variables (eg, hourly vital signs, transfusion responses) may enable adaptive risk stratification.^37^ Finally, patient-centered outcomes, like quality of life or functional status post-bleeding, could align predictions with holistic care goals.^38^^,^^39^ By addressing these gaps, the model can evolve from a predictive tool to a dynamic clinical decision-support system.

## Conclusion

This study introduces and validates a novel ML model for predicting 30-day mortality in GIB using a clustering-based ensemble approach with 16 clinically accessible variables. External validation demonstrated robust performance, significantly outperforming traditional scores. The clustering framework identified distinct patient phenotypes with mortality rates ranging from 0.60% to 15.30%, enabling nuanced risk stratification. SHAP analysis confirmed clinical plausibility, with age, albumin, and markers of organ dysfunction driving predictions. The model's simplicity, interpretability, and open-source adaptability position it for seamless EHR integration. Future prospective validation and incorporation of dynamic variables could transform this tool into a real-time decision aid, advancing personalized care in acute GIB.

## Supplementary Materials

Supplementary Material

Extended PDF
