# Supplementary material for: A Clustering-Based Machine Learning Approach for Mortality Prediction in Gastrointestinal Bleeding: Development and Validation
Source: Gastro Hep Adv. 2026 Apr 24;5(7):100985. doi: 10.1016/j.gastha.2026.100985 (PMC13202545; doi:10.1016/j.gastha.2026.100985)
Supplement: Supplementary Material [file mmc1.pdf]

Supplemental Material

Keyword Screening

Keywords from triage documentation were included if they appeared anywhere in the chief complaint text field. Terms related to GI bleeding identified the primary cohort, from which symptoms of altered mental status were then extracted. After screening, all resulting terms were manually verified for relevance to their respective presentation or symptom. Keyword screening was performed using regular expressions.

Terms used to extract chief complaints related to GI bleeding include: ‘hematemesis’, ‘melena’, ‘hematochezia’, ‘rectal bleed’, ‘gi bleed’, ‘brbpr’, ‘coffee’, ‘bloody stool’, ‘blood in stool’, ‘tarry stool’, ‘blood in vomit’, ‘bloody diarrhea’, ‘ugib’, ‘lgib’, ‘vomiting blood’, ‘gib’.

Terms used to extract symptoms related to altered mental status: 'syncope', 'faint', 'altered', 'unresponsive', 'confused', ‘confusion’, 'lethargy', ‘lethargic’, 'seizure', 'down'.

Identifying patients with GI bleeding from the chief complaints instead of using the ICD codes ensured that patients presented to the ED with GI bleeding rather than developing GI bleeding during hospitalization.

Data Extraction Methods

The MIMIC-IV database is structured into multiple modules, each containing specific data types.

Core Module:

Patient demographics (age, gender) were extracted from the 'patients' and 'admissions' tables

MIMIC-IV-ED Module:

Chief complaints were extracted from the 'triage' table, which contains detailed records of ED visits. Initial vital signs (temperature, blood pressure, heart rate, respiratory rate, oxygen saturation) were retrieved from the 'triage' table

Hosp Module:

Laboratory values were extracted from the 'labevents' table, including:

- Complete Blood Count (CBC): hemoglobin, hematocrit, white blood cell count, platelet count
    - Basic Metabolic Panel (BMP): sodium, potassium, chloride, bicarbonate, blood urea nitrogen, creatinine, glucose
    - Coagulation profiles: prothrombin time (PT), partial thromboplastin time (PTT), international normalized ratio (INR)
    - Liver function tests: albumin, alanine aminotransferase (ALT), aspartate aminotransferase (AST), alkaline phosphatase (ALP), bilirubin
    - Additional tests: lactate, blood gas analysis

Diagnosis codes were extracted from the 'diagnoses_icd' table, which records all ICD diagnoses associated with each hospital encounter. Specific codes used are detailed below.

Data Linkage: MIMIC-IV and MIMIC-IV-ED databases are linked through shared identifiers:

- subject_id: unique patient identifier
- hadm_id: hospital admission identifier
- stay_id: ED stay identifier

This linkage enables patient-level analyses across emergency and inpatient settings, allowing tracking of patients from ED presentation through hospital admission and outcomes.

Diagnostic Codes

Codes below were used to extract co-morbidities from derivation and validation cohorts.

Diagnosis	ICD-9	ICD-10
Acute myocardial infarction	410, 412	I21, I22, I252
Congestive heart failure	398.91, 402.01, 402.11, 402.91, 404.01, 404.03, 404.11, 404.13, 404.91, 404.93, 425.4, 524.5, 425.6, 425.7, 425.8, 425.9	I43, I50, 'I099', 'I110', 'I130', 'I132', 'I255', 'I420', 'I425', 'I426', 'I427', 'I428', 'I429', 'P290'
Liver disease (moderate to severe)	571.2, 571.5, 456.0, 456.1, 456.2, 572.2, 572.3, 572.4, 572.8	K74.02, K74.6, K70.3, K71.7, I85.0, I85.9, I86.4, I98.2, K72.1, K72.9, K76.6, K76.7
Malignancy (excluding skin)	140, 141, 142,143, 144, 145, 146, 147, 148, 149, 150, 151, 152,153, 154, 155, 156, 157, 158, 159, 160, 161, 162, 163, 164, 165, 170, 171, 172, 174, 175, 176, 179, 180, 181, 182, 183, 184, 185, 186, 187, 188, 189, 190, 191, 192, 193, 194, 195, 200, 201, 202, 203, 204, 205, 206, 207, 208, 238.6	C0, C1, C2, C3, C4, C5, C6, C7, C8, C9, C10, C11, C12, C13, C14, C15, C16, C17, C18, C19, C20, C21, C22, C23, C24, C25, C26, C30, C31, C32, C33, C34, C37, C38, C39, C40, C41, C45, C46, C47, C48, C49, 50, C51, C52, C53, C54, C55, C56, C57, C58, C60, C61, C62, C63, C64, C65, C66, C67, C68, C69, C70, C71, C72, C73, C74, C75, C76, C81, C82, C83, C84, C85, C90, C91, C92, C93, C94, C95, C96, C97
Metastatic solid tumor	196, 197, 198, 199	C77, C78, C79, C80

Supplemental Table 1: ICD-9 and ICD-10 codes utilized for diagnoses retrieval from development and validation cohorts.

Model Parameters

K-Means Hyperparameters

n_clusters: 24, init: 'k-means++', n_init: 'auto', max_iter: 300, tol: 0.0001, verbose: 0, random_state: None, copy_x: True, algorithm: 'lloyd'

Random Forest Hyperparameter Search Range

'n_estimators': 50, 100, 200, 300, 400

'max_depth': 3, 5, 7, None

'min_samples_split': 2-11

'min_samples_leaf': 1-10

'bootstrap': True, False

'class_weight': None, 'balanced', 'balanced_subsample'

Final Selected Parameters For Random Forest

Cluster	bootstrap	class_weight	max_depth	min_samples_leaf	min_samples_split	n_estimators
0	TRUE	None	None	2	2	400
1	TRUE	None	None	1	9	200
2	TRUE	None	None	1	9	200
3	TRUE	None	None	1	9	200
4	TRUE	None	None	1	9	200
5	TRUE	None	None	2	2	400
6	FALSE	None	None	2	9	300
7	TRUE	None	7	8	6	100
8	TRUE	balanced	5	2	5	300
9	TRUE	None	None	2	2	400
10	FALSE	None	None	2	9	300
11	TRUE	None	None	1	9	200
12	TRUE	None	None	1	9	200
13	TRUE	balanced	5	2	5	300
14	TRUE	None	7	8	6	100
15	TRUE	None	None	1	9	200
16	FALSE	None	7	5	4	400
17	FALSE	None	None	2	9	300
18	TRUE	None	None	2	2	400
19	FALSE	None	None	2	9	300
20	TRUE	balanced	None	5	3	400
21	FALSE	balanced_subsample	5	5	3	300
22	TRUE	balanced	5	2	5	300
23	TRUE	None	None	1	9	200

Supplemental Table 2: Final hyperparameters chosen for each random forest model after fine-tuning via Randomized Search.

Cluster ID	Count	hematemesis (%)	melena (%)	liver disease (%)	malignancy (%)	heart rate	sbp	hemoglobin	WBC	platelets	bicarbonate	BUN	creatinine	albumin	INR	lactate	age	mortality (%)
4	713	19.8	46	2.2	36.3	75.6	121.72	10.34	10.57	197.9	23.15	24.75	1.32	3.59	1.27	2.88	68.15	2.1
18	592	25.5	54.1	8.1	43.2	77.75	115.34	9.39	11.28	152.25	22.01	30.87	1.63	3.32	1.42	3.06	68.79	13.01
23	555	24	43.1	1.8	37.5	74.78	116.53	10.55	10.76	242	23.2	24.08	1.31	3.7	1.26	2.85	67.54	3.24
13	478	43.1	51.5	31.2	45.6	80.29	124.73	7.81	10.17	42.34	20.15	31.79	1.82	2.81	1.79	4.22	59.59	15.27
10	446	25.6	33.2	0.2	21.3	78.32	118.88	12.67	9.56	277.23	24.24	14.02	1.01	4.17	1.1	2.81	39.13	1.12
2	436	27.8	55.5	8.5	44.5	76.47	138.58	9.51	9.6	123.2	22.34	26.41	1.5	3.29	1.39	2.98	69.85	2.29
6	427	20.8	44.5	0.7	34.9	76.95	117.07	10.55	11.57	323.72	23.17	24.42	1.37	3.69	1.28	2.9	59.63	2.81
9	427	40.5	52.2	21.5	41.7	81.22	116.37	8.94	10.31	91.78	21.07	25.55	1.51	3	1.65	3.57	59.39	9.6
17	411	26.8	36.5	0.7	20.2	89.88	121.65	12.51	9.41	226.21	23.86	15.16	1.02	4.06	1.12	2.86	40.46	2.19
20	337	25.8	45.1	0.6	37.4	77.39	140.33	10.21	12.77	272.2	23.34	25.99	1.36	3.62	1.24	2.71	72.28	0.59
16	317	38.5	35	4.1	23.7	88.51	119.97	11.89	9.23	171.44	23.46	16.03	1.11	3.92	1.2	2.95	41.07	3.79
12	309	20.1	46.9	3.2	35	78.79	150.41	10.32	10.1	175.28	23	26.83	1.58	3.63	1.21	3.04	71.73	0
11	295	19.7	46.8	1.4	33.2	78.17	148.87	10.7	9.57	226.59	23.43	23.46	1.28	3.72	1.24	2.77	69.21	0
1	294	25.9	46.9	1.7	36.4	81.87	124.79	10.27	12.61	371.14	23.34	23.99	1.3	3.67	1.31	2.71	57.04	1.7
22	270	27.4	74.1	10	38.5	79.05	127.52	7.22	12.77	116.25	18.25	94.63	5.01	2.99	1.81	4.03	68.86	7.78
0	208	29.8	61.5	3.4	46.2	80.05	124.16	8.04	15.86	222.67	19.71	98.37	4.91	3.14	1.65	3.26	69.62	7.69
21	199	26.1	50.8	3.5	38.2	79.36	126.19	9.72	13.85	428.11	22.77	30.07	1.49	3.52	1.28	2.74	61.14	1.01
5	156	23.1	46.8	1.3	45.5	79.24	153.28	10.44	12	322.01	23.4	25.01	1.27	3.64	1.33	2.8	68.92	0
15	127	27.6	47.2	1.6	47.2	101.82	117.08	10.46	12.13	291.89	23	25.91	1.31	3.51	1.25	3.17	60.14	10.24
8	98	25.5	50	0	38.8	81.17	125.96	9.18	15.03	507.64	22.8	26.27	1.14	3.41	1.45	2.72	60.96	6.12
19	53	30.2	41.5	0	43.4	82.4	124.74	8.18	17.01	619.68	22.81	25.57	1.24	3.17	1.26	2.7	57.3	1.89
3	15	13.3	66.7	0	26.7	81.47	124	9.02	18.19	801.07	21.4	36.93	1.42	3.25	1.32	3.38	64.93	0
14	3	33.3	66.7	0	100	81.33	121.67	9.03	24.8	1158	22.67	20.33	0.8	3.17	1.11	1.97	64.33	0

Supplemental Table 3: Cluster characteristics.

Internal Validation Metrics

Metric	Ensemble Model (Mean ± 95% CI)	AIMS65 (Mean ± 95% CI)	GBS (Mean ± 95% CI)
AUC	0.8840 (0.8633,0.9046)	0.7094 (0.5995,0.8192)	0.7411 (0.6367,0.8454)
Sensitivity (recall)	0.8680 (0.8266,0.9094)	0.4733 (0.2973,0.6494)	1.0000 (1.0000,1.0000)
Specificity	0.7461 (0.7231,0.7691)	0.8132 (0.7826,0.8437)	0.0785 (0.0481,0.1090)
PPV (precision)	0.1334 (0.1059,0.1609)	0.1032 (0.0643,0.1422)	0.0466 (0.0426,0.0507)
NPV	0.9924 (0.9899,0.9948)	0.9713 (0.9593,0.9832)	1.0000 (1.0000,1.0000)

Comparison of performance metrics between the ensemble model (threshold ≥0.852), AIMS65 (threshold ≥2), and Glasgow-Blatchford Score (GBS, threshold ≥6) on the internal validation cohort. Metrics are presented as mean ± 95% confidence intervals (CIs). AUC: area under the receiver operating characteristic curve; PPV: positive predictive value; NPV: negative predictive value.

Metric	Machine Learning Model (at 0.82 threshold)	AIMS65 (at ≥1 threshold)	GBS (at ≥2 threshold)
Sensitivity	98.30%	91.50%	100%
Specificity	56.10%	35.60%	17.10%
False Positives (FP)	458	672	866
False Negatives (FN)	1	4	0
Positive Predictive Value (PPV)	9.10%	6.00%	5.20%
Negative Predictive Value (NPV)	99.80%	98.90%	100.00%
False-Positive Rate (FPR)	43.90%	64.40%	82.90%

Comparative performance of the machine learning model, AIMS65, and Glasgow-Blatchford Score (GBS) at maximum sensitivity thresholds on the internal validation cohort.
